# Healthcare experiences and barriers as predictors of suicidal thoughts and behaviors among transgender adults: an elastic net regression analysis

**DOI:** 10.1007/s00127-025-02977-2

**Published:** 2025-08-07

**Authors:** Annabelle M. Mournet, Molly I. Ball, Evan M. Kleiman

**Affiliations:** Department of Psychology Rutgers, The State University of New Jersey, Jersey, USA

**Keywords:** Transgender and gender diverse, Suicide, Healthcare barriers

## Abstract

**Purpose:**

Suicide is a critical public health concern, particularly among transgender and gender diverse (TGD) individuals The current study aims to identify structural and interpersonal barriers as well as protective factors that impact suicide risk among transgender and gender diverse adults.

**Methods:**

The currently analyses utilize the TransPop dataset, the first national probability sample of 274 adult transgender adults in the United States. Elastic net regression was used to examine the relationships between the set of 17 healthcare predictor variables and STB outcome variables.

**Results:**

Worries about negative evaluation from healthcare providers emerged as highly important variables across STB outcomes. Inability to access healthcare due to the cost of seeing a doctor was associated with increased suicide plans. Having insurance was associated with decreased odds of suicidal ideation with intent.

**Conclusions:**

There is a need for increased supportiveness and acceptance from healthcare providers as well as a need to ensure that TGD individuals have access to high-quality mental and physical healthcare.

**Supplementary Information:**

The online version contains supplementary material available at 10.1007/s00127-025-02977-2.

## Introduction 

Suicide risk is a critical public health concern, particularly among transgender and gender diverse (TGD) individuals who experience significantly higher rates of suicidal ideation and attempts compared to cisgender individuals [1]. A recent national probability sample highlights these strikingly high rates of suicidal thoughts and behaviors (STBs) among TGD adults: 81.3% of TGD participants reported suicidal ideation in their lifetime and 42.0% reported they had previously attempted suicide [1]. This same study reported that TGD individuals had nearly seven times higher likelihood of having lifetime suicidal ideation and were over four times more likely to have had a lifetime suicide attempt when compared to cisgender adults [1]. These disparately high STB rates among TGD individuals are driven by a variety of social and structural factors, including elevated rates of interpersonal and structural discrimination, limited access to healthcare, mental health comorbidities, and increased involvement in risk behaviors compared to their cisgender counterparts [2–4].

Access to medical and mental healthcare is crucial in efforts to mitigate suicide risk, yet research has specifically highlighted concerns about limitations to access to healthcare among TGD individuals. Transgender adults are both more likely to be uninsured and report cost-related barriers to care compared to cisgender individuals [5]. Additional work to understand healthcare disparities among gender minorities has highlighted that the lack of providers who are sufficiently knowledgeable on healthcare for transgender individuals was the most common barrier cited by transgender individuals [6]. Researchers further highlighted financial barriers, discrimination, cultural incompetence by providers, health systems barriers and socioeconomic barriers as relevant barriers for TGD individuals seeking healthcare [6].

Despite the wealth of literature on barriers to healthcare as well as on suicide risk among the TGD community, there is a gap in the field in understanding whether these experiences are related, namely, how healthcare experiences may be predictive of STBs in the TGD population. The Minority Stress Theory [7] has been posited by many as a framework for understanding how chronic stressors, such as institutional barriers, transphobia, discrimination, and social rejection, contribute to suicide risk in the TGD population as well as healthcare disparities [8, 9]. Applied here, it follows that stressors that manifest within healthcare settings, such as financial constraints, inadequate insurance coverage, or fear of negative evaluation by providers, have the potential to result in poorer mental health outcomes, including a higher risk for suicide.

As one example, a primary barrier to healthcare access among TGD individuals is a lack of insurance or inadequate insurance coverage for gender-affirming care, resulting in high costs of care [10]. Many insurance policies exclude or deny coverage for gender-affirming medical treatments, such as hormone therapy and surgeries, which have been shown to reduce psychological distress and suicide risk significantly [10–12]. Without adequate insurance, transgender individuals often face out-of-pocket costs that make essential healthcare financially inaccessible, exacerbating mental health concerns. Additionally, financial barriers may extend beyond gender-affirming care; the high cost of mental health services if uninsured, particularly therapy with affirming providers, can lead to delays or avoidance of care, further increasing vulnerability to suicidality [10].

For TGD individuals who are able to access healthcare, concerns about negative evaluation and discrimination present another significant barrier. Many transgender individuals report experiencing transphobia, stigma, misgendering, or outright denial of care in medical settings [13, 14]. Anticipatory anxiety about discrimination has been found to result in healthcare avoidance, leaving TGD individuals without necessary medical and psychological support [15, 16]. Research has shown that this avoidance of stigma in healthcare settings is linked to increased odds of depressive symptoms and anxiety symptoms [17].

Healthcare-related barriers may contribute to the disproportionately high suicide risk among TGD individuals, yet research on the specific healthcare variables that are most predictive of STBs remains limited. Leveraging TransPop, the first US national probability survey of transgender adults [18, 19]. Numerous papers to date have leveraged this dataset, including to improve the understanding of suicide risk and healthcare among TGD individuals. For instance, one study examined correlates of non-suicidal self-injury in this population [20]. Other studies using this dataset have looked at an assortment of STB risk factors, such as interpersonal minority stressors, social connection, and demographic risk factors [21, 22]. Feldman and colleagues also highlighted health access disparities for TGD individuals [18] and Strenth and colleagues even examined how access to healthcare relates to suicidal ideation in this population [23]. This paper aims to build upon these existing studies by identifying key healthcare-related variables that act as predictors of STBs in the TGD population. Specifically, an elastic net regression approach is taken as it allows for examination of many variables at once and simplifies models (i.e., reduces dimensionality) to identify which variables among a larger set of possibly related variables are most relevant to the selected outcome. This approach will help to clarify how specific structural and interpersonal barriers within the healthcare system contribute to suicide risk among TGD individuals. We also build upon previous studies by looking at four STB outcomes to understand the specific variables associated with each outcome. With government policies increasingly threatening access to appropriate gender-affirming healthcare and the known mental health risk of discriminatory legislation [24], understanding these relationships is more critical than ever. Identifying healthcare related risk factors can inform targeted interventions, improve healthcare practices, and guide policy efforts to reduce suicide risk in the TGD population.

## Methods

### Study design and participants

This study utilizes the TransPop dataset, which is the first national probability sample of adult transgender individuals in the United States [17, 18]. The goal of this study was to collection large-scale, national information on TGD individuals’ social, economic, physical, and mental health experiences. Data collection occurred from 2016 to 2018. Two different probability sampling approaches were used, namely random digit dialing and address-based sampling. To be eligible, participants had to be 18 years or older, have completed at least sixth grade education, and be able to complete the study interview in English. The overall dataset includes a comparison group of cisgender adults, however the present analyses focus on the 274 TGD participants. A total of 120 participants identified as transgender women (43.8%), 78 identified as transgender men (28.5%), and 76 identified as non-binary (27.7%). The mean age of the sample was 39.4 (SD = 16.9). Regarding age ranges, 27.7% of the sample was ages 18–25 years, 31.8% of the sample was ages 26–40 years, 30.1% of the sample was ages 41–64 years, and 10.6% of the sample was 65 years and older. The sample was predominantly White (68.3%; 187/274). A majority of participants identified as having a sexual minority identity (77.7%; 213/274). This manuscript was exempt from IRB approval due to the use of secondary data exclusively.

## Measures

### Demographics

 Participants responded to demographic questions, including about their gender identity, sexual orientation, age, and race. Table 1 provides demographic information on the participants.

**Table 1:** Demographic and clinical characteristics of sample

Variable	M (SD) / *N* (%)
Age	39.4 (16.9)
18–25 years old	76 (27.7%)
26–40 years old	87 (31.8%)
41–66 years old	82 (30.1%)
65 + years old	29 (10.6%)
Gender	
Transgender woman	120 (43.8%)
Transgender man	78 (28.5%)
Non-binary	76 (27.7%)
Sexual orientation	
Heterosexual	61 (22.3%)
Sexual minority identity	213 (77.7%)
Race/ethnicity	
White	187 (68.3%)
Black	21 (7.7%)
Hispanic	26 (9.5%)
Asian	8 (2.9%)
Native Hawaiian / Pacific Islander	4 (1.5%)
Multiracial	24 (8.8%)
Other	4 (1.5%)
STB outcomes	
Past year suicidal ideation	86 (31.4%)
Past year suicidal ideation with intent	27 (9.9%)
Past year suicide plan	65 (23.7%)
Past year suicide attempt	10 (3.6%)
Healthcare experiences	
Health insurance	250 (91.2%)
Has somewhere to go when sick	181 (66.1%)
Has provider for trans-related healthcare	109 (39.85)
Been to LGBT health clinic in past 5 years	81 (29.6%)
Gender-affirming healthcare	262 (95.6%)
Hormone replacement therapy	131 (47.8%)
Counseling	213 (77.7%)
Counseling for gender identity	138 (50.4%)
Conversion therapy	42 (15.3%)
Cost of doctor prevents healthcare	74 (27.0%)
Self-reported overall health rating	
Excellent health	102 (37.2%)
Average health	103 (37.6%)
Poor health	69 (25.2%)
Healthcare worries*	
Judged	165 (60.2%)
Negative evaluations	160 (58.4%)
Diagnoses negatively affected	143 (52.2%)
Negative stereotypes	103 (37.6%)

Note: *Indicates those who *agreed* or *strongly agreed* with the following items: judged = “When seeking healthcare, I worry about being negatively judged because of my gender identity or sexual orientation”; negative evaluations = “When seeking healthcare, I worry that evaluations of me may be negatively affected by my gender identity or sexual orientation”; diagnoses negatively affected = “When seeking healthcare, I worry that diagnoses of me/my health may be negatively affected by my gender identity or sexual orientation”, negative stereotypes = “When seeking healthcare, I worry that I might confirm negative stereotypes about LGBT people”

### Healthcare

 The TransPop survey items come from an assortment of pre-existing surveys that assess a variety of healthcare experiences and services. Modified items from the National Transgender Discrimination Survey [25] was used to assess over a dozen forms of gender-affirming care (e.g., hysterectomy, phalloplasty, facial surgery), as well as whether they had ever had hormone replacement therapy (HRT), mental health counseling, mental health counseling for gender-related issues, and conversion therapy. Additional questions inquired about current health insurance coverage (modified from the American Community Survey [26] and The Report of the 2015 U.S. Transgender Survey [27]), whether they have somewhere to go when they are sick (National Health Interview Survey [28]), if cost is prohibitive when deciding whether to see a doctor (Center for Disease Control and Prevention Behavioral Risk Factor Surveillance System Survey [29]), whether they have a transgender-related healthcare provider (modified from The Report of the 2015 U.S. Transgender Survey [27]), visits to an LGBT clinic in the past five years (from the Generations Study Baseline Questionnaire and Measure Sources [30]), and a general rating of their own health. Four additional items, based on a stereotype threat measure modified from Abdou and Fingerhut [31], asked participants to rate their level of agreement with a set of statements related to worry about receiving healthcare. For instance: “When seeking healthcare, I worry about being negatively judged because of my gender identity or sexual orientation.” These four items are stated in full in Table 1. **S**

### STB measures

Participants were asked a series of questions to assess history of suicidal ideation, suicidal ideation with intent, suicide plans (includes any thinking about possible means of suicide attempt, even without the presence of a specific plan), and suicide attempts. For each STB construct, participants were also asked their age of when the most recent instance of the experience was (i.e., “About how old were you the last time you made a suicide attempt? Your best estimate is fine.”). This information was used to create a variable representing occurrence of the STB items in the past year. Past year STB outcomes were computed by subtracting the age during which the participant noted the most recent occurrence of the outcome from their reported age. Instances where the difference was 0 or 1 was coded as presence of past year STB outcome and differences of 2 or greater were coded as absence of past year STB outcome.

### Data analysis

Elastic net regression is not well equipped for ordinal, or even multi-categorical data. Accordingly, several variables were recategorized or dummy coded to ensure that variables were all on the same scale. A total of 17 predictor variables were included in the models. We combined all gender-affirming care variables into one, with exception of HRT to reflect the way that these items were asked in the original dataset (HRT asked separately from all other gender-affirming healthcare). The gender-affirming healthcare variables were: top/chest surgery reduction or reconstruction, hysterectomy, clitoral release/metoidioplasty/centurion procedure, phalloplasty, hair removal/electrolysis, breast augmentation/surgery, silicone injections, orchiectomy, vaginoplasty/labiaplasty/sexual reassignment surgery/gender reassignment surgery/gender confirmation surgery, trachea shave, facial feminization surgery, voice therapy, voice surgery, and other. Several categorical predictor variables were made into binary predictors. Based on a bell curve distribution, dummy coding was used to make the rating of overall health, which was originally a categorical variable with five options ranging from “poor” to “excellent” into three separate binary variables of poor, average, and excellent health. Additional variables were recoded to combine response options based on response distributions. Response options for visiting an LGBT clinic in the past five years were “often”, “sometimes,” and “never”; “often” and “sometimes” were combined to reflect having been to an LGBT clinic in the past five years. Healthcare worry items utilized a 5-point Likert scale from “strongly disagree” to “strongly agree”. Responses were recoded to combine “strongly disagree”, “disagree”, and “neutral” into one option and “agree” and “strongly agree” were combined as well. Raw data for recoded items are reported in Supplement A.

Elastic net regression was used to examine the relationships between 17 healthcare-related predictor variables and STB outcome variables. This approach allows us to examine the impact of many variables at once and ultimately reduce the oversized impact of some variables while also eliminating those that did not contribute meaningful information. Elastic net regression is a valuable approach when seeking to understand which variables, among a larger set of variables are most important. A separate elastic net model was fitted for each STB outcome to account for potential differences in predictor-outcome associations. For each model an indicator of the relative importance of each variable was computed by standardizing the regression coefficients. Relative importance values range from 0 to 100, where variables with a relative importance score of 50 are half as strong predictors of the given outcome as a variable with a relative importance score of 100. Variables with a relative importance score of 50 or greater were retained in the model. For models where less than three variables were above 50, the top three variables were retained. The elastic net models were run using the *glmnet* package in R with the selected variables included [32].

Elastic net regression differs from traditional inferential statistical methods in that it emphasizes coefficient regularization to improve predictive performance and reduce overfitting. As a result, standard p-values and confidence intervals are not statistically valid due to the bias introduced by penalization and the effects of variable selection [33, 34]. Instead, model performance metrics, such model accuracy rate, are typically reported. The model accuracy rate was calculated for each model, which refers to how well the model predicts the outcome variable. The accuracy of each model was tuned by adjusting the regularization parameters of alpha (α) and lambda (λ). Odds ratios are reported for each predictor variable for each model. Odds ratios reflect associations adjusted for the other predictors in the models. Of note, we refer to variables as predictor variables. Though “predictor” is often reserved for longitudinal research [35], in this cross-sectional study, “predictor” is used to reflect the items that are part of the model building, as opposed to causal inference, as is consistent with existing literature on model building, including in cross-sectional research [e.g., [36]].

Elastic net regression requires complete data for all variables included in the model. We elected to not impute data due to the relatively small amount of missing data. Across 274 participants, 257 had complete data, which computes to 6.2% missing data (7.9% missing data in training data and 3.6% missing data for test data).

## Results

### Descriptive statistics

Past year suicidal ideation was reported by 31.4% of participants (86/274) and 9.9% of participants reported past year suicidal ideation with intent (27/274). Regarding suicide plans, 23.7% (65/274) reported past year suicide plans and 3.6% of participants (10/274) reported attempting suicide in the past year. A majority of participants reported having health insurance (91.2%; 250/274). Approximately two-thirds of participants reported having somewhere to go when sick (66.1%; 181/274), 39.8% (109/274) reported having a provider for transgender-related healthcare, and 29.6% of participants (81/274) reported having been to an LGBT-specific health clinic in the past five years. Participants lived an average of 67.3 miles from an LGBT health center (SD = 235.1). Most participants reported having used some form of gender-affirming healthcare (95.6%; 262/274), with 47.8% specifically reporting use of HRT (131/274). Over three-quarters of participants (77.7%; 213/274) reported having been to therapy before, with 50.4% of participants (138/274) reporting having been to counseling specifically related to gender identity previously. Table 1 reports STB and healthcare information to further characterize the sample. 

### Elastic net regressions

#### Suicidal ideation

**Fig. 1:**
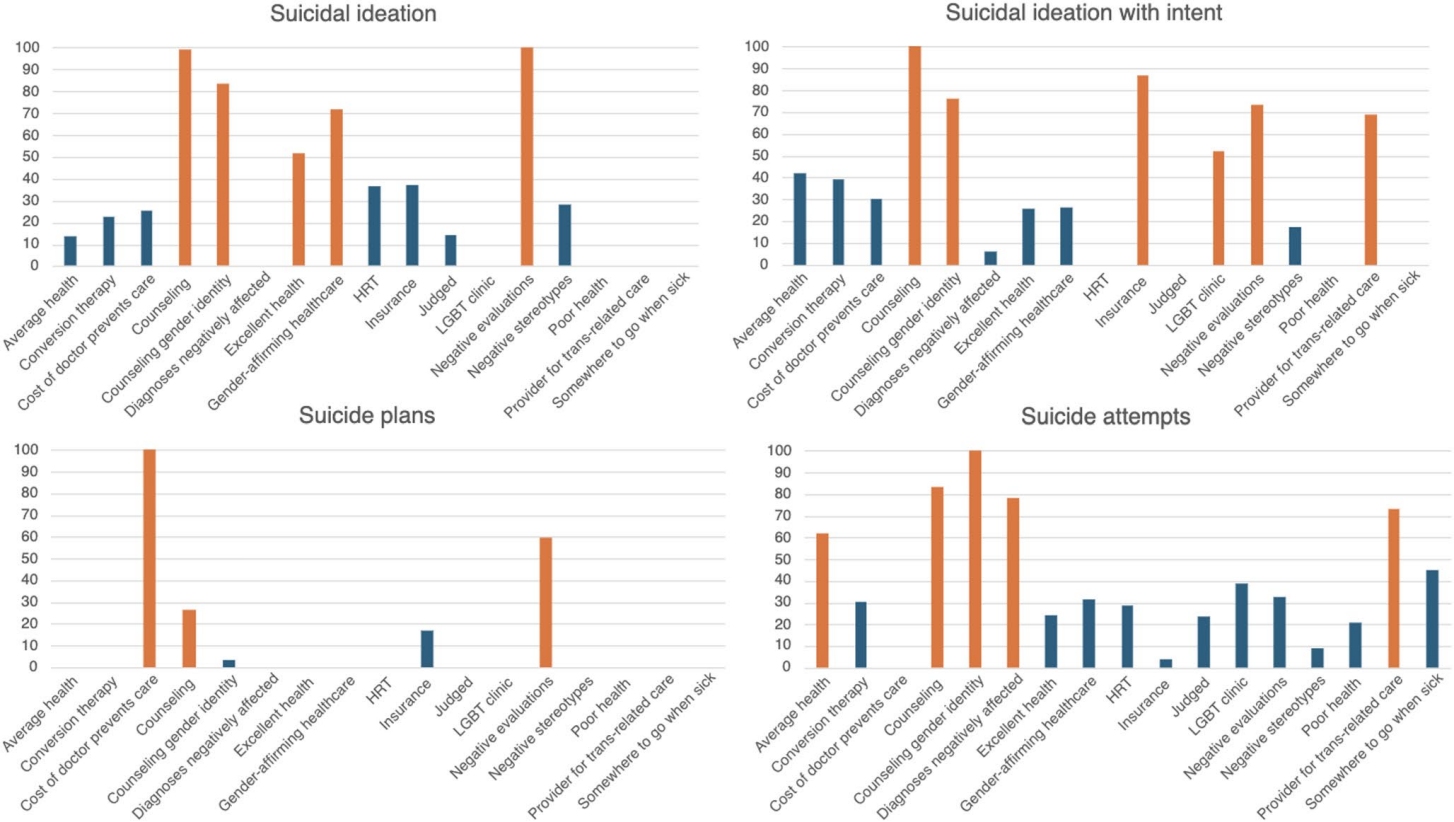
Variable importance across STB outcomes. Note: Orange = variable included in elastic net regression

The suicidal ideation model had a model accuracy rate of 71.8%. The variables with the greatest importance were worry when seeking healthcare that evaluations from others may be negatively affected by gender identity or sexual orientation (variable importance = 100), counseling (variable importance = 99), counseling related to gender identity (variable importance = 83.5), gender-affirming healthcare (variable importance = 71.9), and self-reported excellent health (variable importance = 51.73). Participants with worries about evaluations (OR = 2.45), those who had received counseling related to gender identity (OR = 2.01), or had counseling broadly (OR = 1.97), and those with gender-affirming healthcare (OR = 1.60) had higher odds of suicidal ideation. Participants with self-reported excellent health had lower odds of suicidal ideation (OR = 0.57). Figure 1 depicts variables with greatest importance and associated odds ratios for all STB outcomes.

#### **Suicidal ideation with intent**

The suicidal ideation intent model had a model accuracy rate of 91.8%. The variables of greatest importance were counseling (variable importance = 100), insurance (variable importance = 86.7), counseling related to gender identity (variable importance = 76.1), worry when seeking healthcare that evaluations from others may be negatively affected by gender identity or sexual orientation (variable importance = 73), having a provider to go to for transgender-related healthcare (variable importance = 68.9), and having been to an LGBT-specific health clinic (variable importance = 51.7). Participants with worries about evaluations (OR = 1.78), those who have received counseling (OR = 1.72), those who have received counseling related to gender identity (OR = 1.66), and individuals who had been to an LGBT-specific health clinic (OR = 1.28) had higher odds of past year suicidal ideation with intent. Participants with insurance (OR = 0.53) and those with a provider to go to for transgender-related healthcare (OR = 0.73) had lower odds of past year suicidal ideation with intent. 

#### Suicide plans

The suicide plans model accuracy rate was 70.0%. Variables with the greatest importance were needing to see a doctor in past year but could not due to the cost (variable importance = 100), worry when seeking healthcare that evaluations from others may be negatively affected by gender identity or sexual orientation (variable importance = 59.5), and counseling (variable importance = 26.6). Participants who could not see a doctor in the past year due to cost (OR = 2.28), those with worries about negative evaluations (OR = 1.71), and those who have received counseling (OR = 1.44) had higher odds of suicide plans in the past year.

#### **Suicide attempts**

The model accuracy rate for the suicide attempts model was 97.3%. The variables with greatest importance were counseling related to gender identity (variable importance = 100), counseling (variable importance = 83.3), worry when seeking healthcare that diagnoses may be negatively affected by gender identity or sexual orientation (78.2), having a provider to go to for transgender-related healthcare (variable importance = 73.1), and self-reported good health (variable importance = 62.1). Participants with worries about diagnoses being impacted by gender identity or sexual orientation (OR = 1.29), those who had received counseling related to gender identity (OR = 1.26), or had counseling broadly (OR = 1.22), and individuals with self-reported average health (OR = 1.21) had higher odds of suicide attempts in the past year. Participants with a provider to go to for transgender-related healthcare (OR = 0.91) had lower odds of past year suicidal ideation.

## Discussion

Through the use of elastic net regression, the present analyses revealed several variables that were most relevant in determining whether a TGD participant had suicidal thoughts, plans, and attempts. Worries about being negatively evaluated by one’s healthcare provider or having diagnoses impacted due to gender identity or sexual orientation emerged as highly important variables across STB outcomes. Results further highlighted that individuals who have received counseling have elevated odds of STB outcomes; it appears that the individuals who most need counseling to address STB risk are being correctly identified and treated. Additional findings provided further valuable directions to improve suicide prevention approaches among TGD individuals, with an emphasis on targeting healthcare experiences.

An important and consistent finding was the impact of worries about being negatively evaluated by one’s healthcare provider or having diagnoses impacted due to gender identity or sexual orientation on STB outcomes in this population. This underscores the need for clear demonstration of acceptance and support from healthcare providers, including through the use of correct pronouns, avoiding gendered language when describing health conditions and treatments, as well as educating oneself and transgender health topics to remove the burden from patients on needing to educate their providers [37]. Numerous existing studies have highlighted specific approaches that healthcare providers can take to demonstrate their support and improve their care of TGD patients [37, 38]. This is further supported by the finding that individuals with a provider they could go to for transgender-related healthcare had lower odds of suicidal ideation with intent and suicide attempts. Additionally, worries about negative evaluation and experiences of transphobia and discrimination may be a result of negative experiences in participants’ daily lives with non-providers, which extend to fears in the healthcare system. This information may be relevant to other countries, beyond the United States, where gender-affirming treatments may be covered more consistently by insurance. High-quality gender-affirming care, goes beyond insurance, prescriptions, and diagnoses. It necessitates active work to maintain a positive patient-provider relationship, where the patient feels supported.

Despite results underscoring the importance of supportive and accepting providers, some additional results appeared to be somewhat in contrast. Individuals who reported having been to an LGBT clinic in the past year as well as individuals who had gender-affirming care had higher STB risk, specifically suicidal ideation with intent and suicidal ideation, respectively. Importantly, the items pertaining to worries about negative evaluations and diagnoses being impacted as well as having a transgender-related healthcare provider were asked about the present, whereas going to an LGBT clinic was specific to the last five years and having gender-affirming healthcare was about lifetime experiences. Accordingly, findings of STB risk among those who reported having been to an LGBT clinic in the past 5 years and having ever had gender-affirming care may be reflective of current distress and gender dysphoria, which is linked to increased suicide risk [39], or it could be reflective of previous experiences of gender dysphoria. Thus, these findings could reflect an artifact of participants who previously had experience greater levels of risk at an earlier timepoint. Given the variation in the timescales used, future research would benefit from examining the relationship between these risk factors and STB outcomes using a more intensive study design that allows for improved understanding of the temporal nature of these relationships.

Receiving mental health counseling was the only variable that was consistently associated with increased odds of STBs, across each outcome. It is to be expected that those receiving counseling, or those have previously, would be the individuals with greatest STB risk. While the aim of therapy is to reduce suicide risk, these findings suggest that the individuals who are most in need of counseling are receiving that support. Counseling for gender identity-related concerns also emerged as an important variable. Given the impact of gender dysphoria on STB risk [39]. this is also to be expected. These findings suggest the need for continued identification and treatment of TGD adults who are likely to be at increased risk of mental health challenges [2], given the considerable discrimination and stressors experienced by this population [3].

Ability to access healthcare was also associated with STB risk in this population. Inability to access healthcare due to the cost of seeing a doctor was associated with higher suicide plans. Moreover, having insurance was associated with lower odds of suicidal ideation with intent. These findings support the notion that inability to obtain healthcare due to financial challenges impacts suicide risk among TGD individuals. Accordingly, legislation is needed to support the ability of TGD individuals to access affordable healthcare. Notably, self-reported excellent health was associated with lower odds of suicidal ideation, which is consistent with previous research showing medical problems to be a suicide risk factor [40]. Ensuring that TGD individuals have access to healthcare, in order to promote excellent health, is a valuable form of suicide prevention in this population.

Notably, average health was found to be associated with higher odds of suicide attempts, whereas poorer health was associated with higher odds of suicidal ideation and suicide plans, highlighting the unique relationships between healthcare variables and the STB outcomes. One potential mechanistic explanation for this finding related to specific health rating and associated STBs relates to the idea of acquired capability [41]. While individuals with poorer health may have suicidal ideation and plans, it is possible that in a poor health state they lack the functional capacity to act on suicidal urges. Thus, while poor health is a known suicide risk factor [42], it may not translate into attempts due to barriers to enact plans. Comparatively, individuals with average health have underestimated distress from appearing functionally capable but perhaps still have unmet health needs and/or chronic conditions, leading to a nuanced position wherein distress is present, and they have the physical capability to act on suicidal urges, leading to higher attempt rates. The current findings underscore the importance of considering each STB outcome as distinct to understand the factors associated with each outcome. Such delineation of STB variables may allow for improved understanding, prediction, and prevention of such outcomes.

It is important to consider the current findings within in the context of the following limitations. Most notably, this study was cross sectional in design, preventing analyses that parse apart temporal associations. Future studies should employ longitudinal designs to better identify healthcare risk and protective factors for STBs in this high-risk population. For instance, research studies will be needed to understand the long-term impact of medications taken as part of gender-affirming care [43]. Additionally, on a smaller timescale, leveraging ecological momentary assessment would allow for identification of proximal risk and protective factors, allowing for clear intervention targets to reduce suicide risk for TGD individuals. While this study was cross sectional, numerous time scales were used across the items, including lifetime, past five years, as well as the present moment. The use of different time scales further complicates some of the findings, again suggesting the value of an intensive longitudinal design that allows for improved understanding of temporality. Finally, to optimally leverage elastic net regression, many variables were recategorized or dummy coded, which may impact power.

This study extended upon previous research using the TransPop database and revealed several healthcare-related variables that were most impactful on STB risk. The current findings support the need for healthcare providers to be supportive and accepting as well as to ensure that TGD individuals are able to access high-quality mental and physical healthcare. Access to such care is essential for preventing suicide among this population.

## Supplementary Information

Below is the link to the electronic supplementary material.

Supplementary file 1 (docx 35901 KB)

## Data Availability

The data used in this study is publically available: Meyer, Ilan H. TransPop, United States, 2016-2018. Inter-university Consortium for Political and Social Research [distributor], 2021-06-23. https://doi.org/10.3886/ICPSR37938.v1.
