# Supplementary material for: Healthcare experiences and barriers as predictors of suicidal thoughts and behaviors among transgender adults: an elastic net regression analysis
Source: Soc Psychiatry Psychiatr Epidemiol. 2025 Aug 7;61(3):525–33. doi: 10.1007/s00127-025-02977-2 (PMC12995987; doi:10.1007/s00127-025-02977-2)
Supplement: Supplementary file 1 — (docx 35901 KB) [file 127_2025_2977_MOESM1_ESM.docx]

Supplement A: Raw data for recoded items

| **Variable** | **Raw data** |
| --- | --- |
| Gender-affirming healthcare *(N refers to those who responded “Have had it” for each of the items)* | Top/chest surgery reduction or reconstruction: N=35  Hysterectomy: N=20  Clitoral release/metoidioplasty/centurion procedure: N=2  Phalloplasty: N=0  Hair removal/electrolysis: N=57  Breast augmentation/surgery: N=18  Silicone injections: N=3  Orchiectomy: N=23  Vaginoplasty/labiaplasty/sexual reassignment surgery/gender reassignment surgery/gender confirmation surgery: N=17  Trachea shave: N=7  Facial feminization surgery: N=13  Voice therapy: N=20  Voice surgery: N=2  Other: N=11 |
| Overall health rating | Poor: N=19  Fair: N=50  Good: N=103  Very good: N=74  Excellent: N=28 |
| Visting an LGBT clinic in the past 5 years | Often: N=31  Sometimes: N=50  Never: N=141 |
| Healthcare worry items - Judged | Strongly disagree: N=31  Disagree: N=41  Neutral: N=35  Agree: N=91  Strongly agree: N=74 |
| Healthcare worry items – Negative evaluations | Strongly disagree: N=30  Disagree: N=43  Neutral: N=38  Agree: N=94  Strongly agree: N=66 |
| Healthcare worry items – Diagnoses negatively affected | Strongly disagree: N=31  Disagree: N=49  Neutral: N=49  Agree: N=80  Strongly agree: N=63 |
| Healthcare worry items – Negative stereotypes | Strongly disagree: N=63  Disagree: N=62  Neutral: N=44  Agree: N=61  Strongly agree: N=42 |
